# Low-Cost, High-Performance Fiber Optic Fabry–Perot Sensor for Ultrasonic Wave Detection

**DOI:** 10.3390/s19020406

**Published:** 2019-01-19

**Authors:** Haoyong Li, Delin Li, Chaoyu Xiong, Wenrong Si, Chenzhao Fu, Peng Yuan, Yiting Yu

**Affiliations:** 1 Key Laboratory of Micro/Nano Systems for Aerospace (Ministry of Education), Northwestern Polytechnical University, Xi’an 710072, China; lyhaoyong@mail.nwpu.edu.cn (H.L.); delinli@mail.nwpu.edu.cn (D.L.); bearluckyyeah@163.com (C.X.); 2 Key Laboratory of Micro- and Nano-Electro-Mechanical Systems of Shaanxi Province, Northwestern Polytechnical University, Xi’an 710072, China; 3 State Grid Shanghai Electric Power Research Institute, Shanghai 200437, China; siwenrong@126.com (W.S.); 13512111246@139.com (C.F.); 4 Xi’an Maorong Power Equipment Co., Ltd., Xi’an 710048, China; y5anpeng@126.com

**Keywords:** ultrasonic pressure detection, Fabry–Perot, fiber optic sensors

## Abstract

This study describes a novel fiber optic extrinsic Fabry–Perot interferometric (EFPI) ultrasonic sensor comprising a low-cost and high-performance silicon diaphragm. A vibrating diaphragm, 5 μm thick, was fabricated by using the Microelectromechanical Systems (MEMS) processing technology on a silicon-on-insulator (SOI) wafer. The Fabry–Perot (FP) cavity length was solely determined during the manufacturing process of the diaphragm by defining a specific stepped hole on the handling layer of the SOI wafer, which made the assembly of the sensor easier. In addition, the use of cheap and commercially available components and MEMS processing technology in the development of the sensing system, limited the cost of the sensor. The experimental tests showed that the minimum detectable ultrasonic pressure was 1.5 mPa/sqrt(Hz) −0.625 mPa/sqrt(Hz) between 20 kHz and 40 kHz. As a result, this sensor has the potential to successfully detect weak ultrasonic signals.

## 1. Introduction

Ultrasonic waves (frequency >20 kHz) have extensive applications in diverse areas, such as detection of defects in various materials and structures, sonar detection, medical diagnostics, etc. In these applications, ultrasonic sensors always play a vital role in acquiring ultrasonic wave information. The fiber optic extrinsic Fabry–Perot interferometric (EFPI) sensors have a number of inherent advantages such as; high-frequency response, immunity to electromagnetic interference (EMI), remote sensing capability, small size, light weight and easy construction; therefore, they have become an ideal candidate for ultrasonic detection [1,2,3,4,5].

The diaphragm is one of the core components of optical fiber EFPI sensors. To ensure that it has an efficient frequency response to ultrasonic waves, the diaphragm is generally designed to have a high natural frequency, which results in a low sensitivity. For this reason, the trade-off between the natural frequency and the sensitivity must be considered, and the material and dimension of a diaphragm need to be optimized in order to obtain a desired sensitivity as well as the required natural frequency [6]. Wonuk Jo et al. fabricated an EFPI acoustic sensor using a photonic-crystal silicon diaphragm with a thickness of 450 nm. The sensor showed very high sensitivity and excellent minimal detectable pressure, but its maximal detectable acoustic frequency was relatively low [2]. Lijun Song et al. proposed an optical fiber EFPI ultrasonic sensor whose 150 μm-thick fused silicon diaphragm can detect over 100 kHz ultrasonic waves, but its sensitivity is greatly reduced [7].

At present, there are numerous methods to construct a Fabry–Perot (FP) cavity on the end surface of an optical fiber [6,7,8,9,10]. The traditional method consists in adjusting the distance between the fiber and the diaphragm by using a precise positioning platform under the continuous monitoring of a spectrometer and in performing bonding packaging when the cavity length is at the working point [8]. This method can assemble the FP cavity precisely but it is inefficient. Another common method involves adjusting the central wavelength of the light source by an optical filter to ensure the desired FP cavity length [9]. Currently, researchers tend to determine the FP cavity length during diaphragm fabrication in order to improve the efficiency of assembly [10,11,12,13,14]. E. Cibula et al. proposed a new all-glass fiber EFPI pressure sensor. An air cavity on the fiber was formed firstly by an etching process, and then the air cavity was fused with a lead-in fiber to form the FP cavity [10]. The Stanford group described an EFPI acoustic sensor, where the length between the optical fiber and the diaphragm was controlled by attaching two thin spacers to the fixed fiber capillary [11]. These methods can realize the precise assembly of the FP cavity on optical fiber. However, it is urgent to further facilitate sensor assembly and simplify the process. In addition, in most of the reported work, the optical fiber must be preprocessed and fixed by capillaries before sensor assembly, which remains a barrier for the mass production of the optical fiber EFPI sensors [8,9,10,11,12].

In this research, the vibrating diaphragm of a sensor with a thickness of 5 μm was fabricated by employing the Microelectromechanical Systems (MEMS) processing technology on a silicon-on-insulator (SOI) wafer. A stepped hole was designed on the handling layer of the SOI wafer to define the FP cavity when manufacturing the diaphragm, which made the sensor assembly easier. In addition, a commercially available optical fiber patch cord (PC) was directly employed to assemble the FP cavity without any follow-up processing. The experimental test showed that the minimum detectable ultrasonic pressure of the sensor was from 1.5 mPa/sqrt(Hz) to 0.625 mPa/sqrt(Hz) between 20 kHz and 40 kHz.

## 2. Design and Fabrication

### 2.1. Structural Design

**Figure 1:**
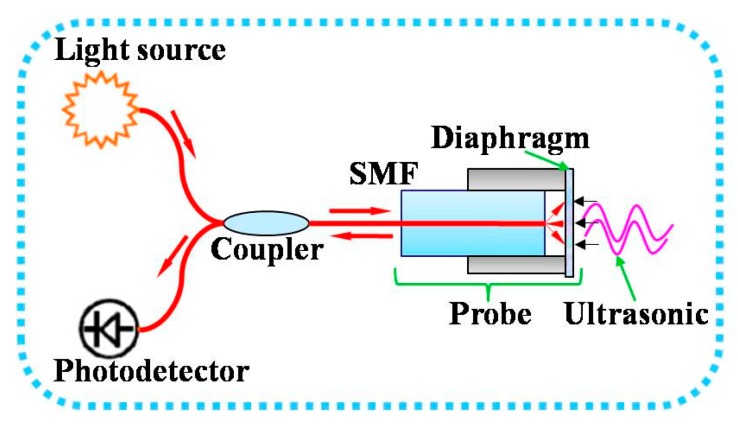
Schematic illustration of the fiber optic extrinsic Fabry–Perot interferometric (EFPI) ultrasonic sensor.

The EFPI sensor was interrogated by using the quadrature detection technique [15], which uses a laser source at a fixed wavelength and a photodetector, as shown in Figure 1.

The ultrasonic wave is a dynamic excitation source. To ensure the response of the EFPI sensor to the ultrasonic wave, the natural frequency *f* of the vibrating diaphragm must be determined first during the design, and that of the circle diaphragm can be expressed by Equation (1), according to reference [16]:

(1) $$f=\frac{ah}{4\pi R^{2}}\sqrt{\frac{E}{3\rho (1-\mu^{2})}}$$

where *a* is the coefficient of vibration mode, *ρ*, *E*, *μ*, *h*, and *R* are density, elastic modulus, Poisson’s ratio, thickness, and radius of the diaphragm, respectively.

After the natural frequency was determined, the larger central deformation *y*(*p*) of the diaphragm corresponded to the higher sensitivity under the same acoustic pressure *p*. Since the ultrasonic wave is a dynamic signal, the diaphragm deformation depends on the damping ratio and its stiffness. In practice, static pressure is usually applied to replace the dynamic pressure during diaphragm design, since the damping ratio and stiffness are difficult to determine. When the static pressure *p* is applied on the circle diaphragm, its central deformation *y*(*p*) can be obtained by Equation (2) [16]:

(2) $$y(p)=\frac{3(1-\mu^{2})p}{16Eh^{3}}R^{4}$$

As a result, after the natural frequency of the diaphragm that is necessary to achieve the maximal deformation has been determined, only the material and geometrical parameters (*R* and *h*) of the diaphragm need to be optimized.

If the material and the natural frequency of the diaphragm are determined, the ratio *h*/*R*^2^ is a constant according to Equation (1), and thus *R* and *h* must increase or decrease at the same time to determine the maximum diaphragm deformation. However, referring to Equation (2), when *R* and *h* increase or decrease at the same time, the change of diaphragm deformation *y*(*p*) is still uncertain. For the purpose of solving the above problem, we can multiply Equations (1) and (2), obtaining Equation (3):

(3) $$y(p)=\frac{1}{f}\cdot \frac{ah}{4\pi R^{2}}\sqrt{\frac{E}{3\rho (1-\mu^{2})}}\cdot \frac{3(1-\mu^{2})p}{16Eh^{3}}R^{4}=\frac{ap}{4\pi f}\cdot \sqrt{\frac{E}{3\rho (1-\mu^{2})}}\cdot \frac{3(1-\mu^{2})}{16E}\cdot (\frac{R}{h}{)}^{2}$$

According to Equation (3), the conclusion can be drawn that when the material and the natural frequency of the diaphragm are determined, the larger the ratio of *R*/*h* is, the greater the diaphragm deformation will be under the same acoustic pressure *p*. In our previous research, we concluded that once the natural frequency and the material of the diaphragm are fixed, the smaller the thickness *h¡*, the larger the *R*/*h* ratio [16]. In other words, if the natural frequency of the diaphragm has been determined, a thinner diaphragm will result in a larger deformation.

**Figure 2:**
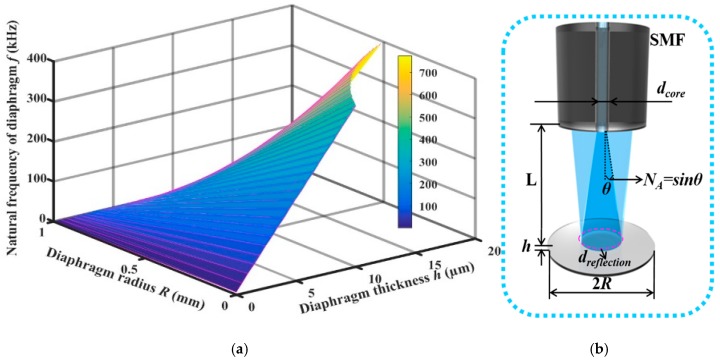
(**a**) Natural frequency versus thickness *h* and radius *R* of the diaphragm, (**b**) Schematic diagram for a fiber optic extrinsic Fabry–Perot (FP) interferometer.

However, by calculating the natural frequency of the diaphragm versus the thickness *h* and the radius *R* according to Equation (1), as shown in Figure 2a, we found that when the natural frequency of the diaphragm is invariable, the radius *R* decreases rapidly with the decreasing thickness *h*. When *R* is too small, sensor assembly is very difficult. Moreover, for fiber optic EFPI sensors, the diaphragm size must be larger than that of the reflecting area for FP interference. This diameter can be calculated by the formula: *d_reflection_* = *d_core_* + 2*N_A_L* (*d_reflection_*, *L, d_core_*, *N_A_* are the diameter of the reflecting area, the FP cavity length, the core’s diameter, and the numerical aperture of the fiber optic, respectively), as denoted in Figure 2b. Therefore, although the deformation of the diaphragm will increase as the diaphragm thickness decreases, the diaphragm thickness cannot be reduced infinitely, considering the sensor assembly and the reflecting area. Currently, most of the reported silicon diaphragm are much larger than 20 µm, which is mainly attributed to the limitation of micromachining processes. Therefore, reducing the diaphragm thickness is still the primary way to improve the performance of fiber optic EFPI ultrasonic sensors.

**Figure 3:**
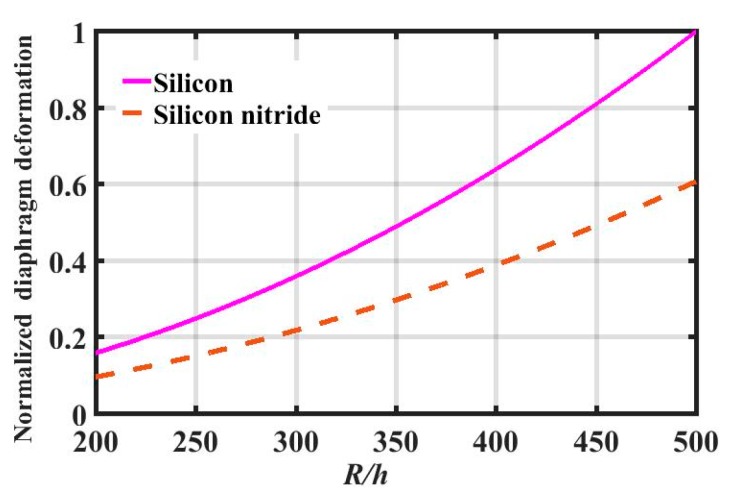
Normalized central deformation *y*(*p*) of silicon and silicon nitride diaphragms at different *R*/*h* ratios.

Silicon and silicon nitride are commonly chosen as diaphragm materials for fiber optic EFPI sensors because of the mature related processing technology and good mechanical behavior. The normalized central deformation *y*(*p*) (deformation of silicon and silicon nitride divided by the maximum deformation of silicon) of these two types of diaphragm at different *R*/*h* ratios can be calculated according to Equation (3), as shown in Figure 3, where the natural frequency of the diaphragm and the acoustic pressure are assumed to be 1. The characteristic parameters of silicon and silicon nitride used in the calculation are given in Table 1. The result shows that the central deformation of a silicon diaphragm is larger than that of a silicon nitride diaphragm for a specific *R*/*h* ratio. Consequently, referring to the conclusion mentioned above, when the natural frequency is established, for the same diaphragm size, the deformation of a silicon diaphragm is larger than that of a silicon nitride diaphragm under the same acoustic pressure.

As a result, in this research, a silicon diaphragm with the natural frequency of 60 kHz was designed by employing ANSYS (Ver. 14.5), since the ultrasonic wave in this frequency is widely applied in the industry, defense, and biomedical fields. The diaphragm thickness was 5 µm, and the diameter was 1120 µm. In order to accurately control the diaphragm thickness, the MEMS processing technology was applied to a SOI wafer. To define the cavity length during the fabrication of the diaphragm, a deep hole and a shallow hole with different diameters were etched in the handling layer of the SOI wafer, generating a stepped hole with step width of 690 µm, to accurately position the single mode fiber (SMF). In addition, a commercially available optical fiber PC was employed to replace the customized polished fiber that is often used in most studies.

### 2.2. Fabrication

**Figure 4:**
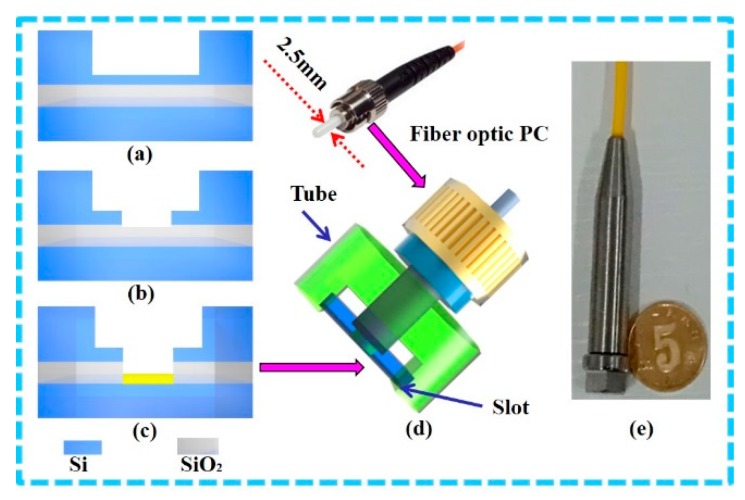
Schematic of fabrication and assembly process of the fiber optic EFPI sensor: (**a**) Etching of a deep hole on the handling layer of the silicon-on-insulator (SOI) wafer, (**b**) Etching of a smaller hole in the bottom of the previous deep hole, (**c**) Removal of the oxide layer and sputtering of the gold film, (**d**) Assembly of the sensor probe, (**e**) Optical image of the sealed EFPI sensor.

The fabrication process of the fiber optic EFPI sensors is illustrated in Figure 4. First, a deep hole with diameter of 2.5 mm and depth of 470 μm was etched on the handling layer of the SOI wafer (device layer 5 μm, box layer 1 μm, and handling layer 500 μm, commercially available from Ultrasil Co.) by using a deep reactive ion-etching (DRIE) process (Figure 4a). Then, a smaller hole with diameter of 1120 µm and depth of 30 μm was etched at the bottom center of the previous deep hole using the spray coating, photolithography, and DRIE processes (Figure 4b). Next, the oxide layer at the bottom of the small hole was completely etched using the buffered oxide etching (BOE) process, and the device layer was used as the self-stopping layer. Finally, a 100 nm-thick gold film was sputtered onto the inner surface of the device layer, behaving as a reflective surface of the FP cavity (Figure 4c). Afterwards, a tube with a slot, functioning as a positioning component for the diaphragm, was manufactured using the 3D printing technology. During the assembly procedure, this tube was fixed in the slot, and the fiber optic PC (type: straight tip, corning: SMF-28e, outside diameter: 2.5 mm) was inserted into the tube and then in the deep hole of the diaphragm (Figure 4d). Finally, the fiber optic PC was fixed by using epoxy (353ND) (commonly employed for its long-time reliability). The fabricated sensor probe is shown in Figure 4e.

**Figure 5:**
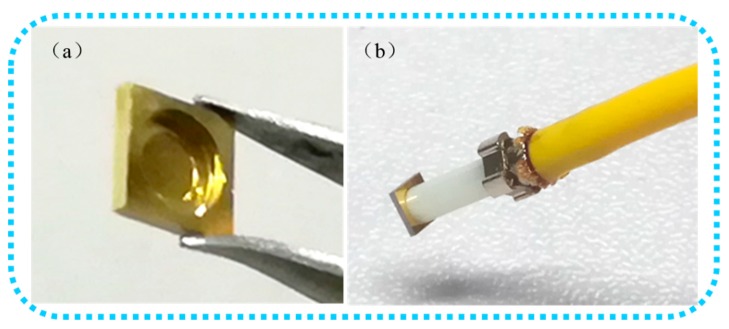
(**a**) Final processed diaphragm, (**b**) Final probe (removed from the protection structure).

This procedure presents several advantages: (1) High sensitivity: The thinner the diaphragm thickness is, the higher its sensitivity will be. Therefore, a 5 μm-thick silicon diaphragm ensures a higher sensitivity than the one obtained so far with other procedures; (2) Low cost: Benefitting from the MEMS fabrication technology, the overall cost of the sensor probe is relatively low, because a single 4 inch SOI wafer can contain hundreds of diaphragms (≈400, each diaphragm measuring 3.5 × 3.5 mm^2^ after scribing). Moreover, the simplified fabrication process can further reduce the cost of the sensor; (3) Easy assembly: The FP cavity length is determined by the DRIE and BOE processes, which re controlled by adjusting the process parameters during the first production. The final processed diaphragm and probe (removed from the protection structure) are shown in Figure 5. The stepped hole determines the actual position of the fiber optic PC in the axial direction, defining the cavity length as needed, and thus the assembly procedure is easier and simpler.

## 3. Experimental Validation

### 3.1. Initial FP Cavity Length

**Figure 6:**
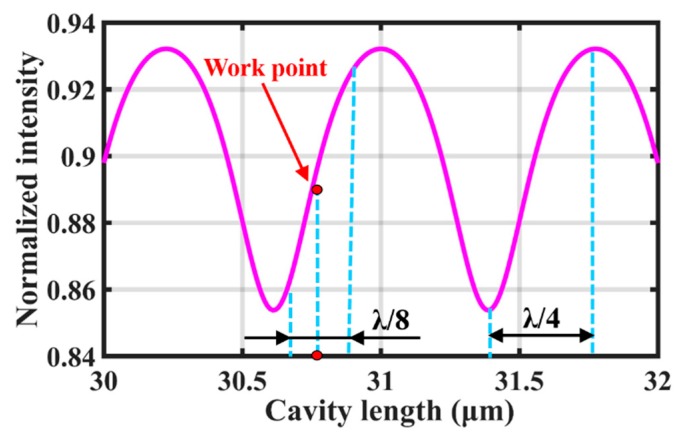
Relationship between normalized intensity and cavity length.

**Figure 7:**
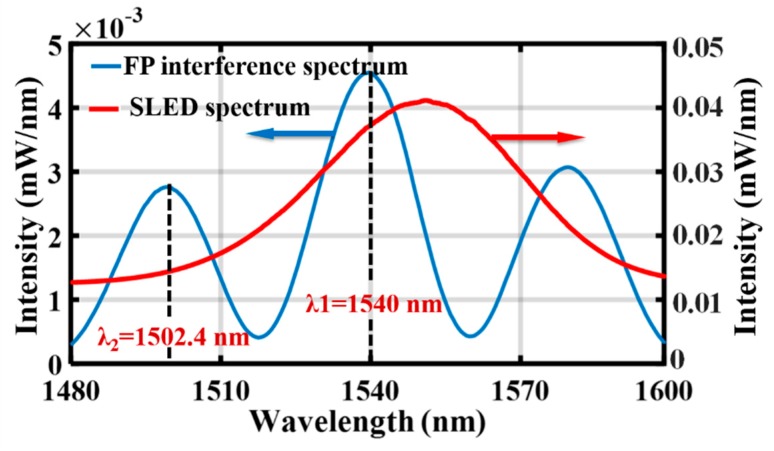
Interference spectrum of the FP cavity together with the superluminescent light emitting diode (SLED) spectrum.

Figure 6 shows the relationship between normalized intensity and cavity length, as well as the working point designed for the EFPI sensor, which was calculated by the output intensity *I*_1_ of the FP interference according to Equation (4):

(4) $$I_{1}=I_{0}(R_{1}+R_{2}+2\sqrt{R_{1}R_{2}}\cos\phi )$$

where *I*_0_ is the intensity distribution of the light source, *R*_1_ and *R*_2_ represent the reflectivity of the optical fiber end surface and the internal face of diaphragm, and *ϕ* is the round-trip phase shift, given by *ϕ* = 4π*d*/*λ* (*d* is the FP cavity length, and *λ* is the central wavelength of the light source), and thus the accuracy of the initial cavity length must be at the nanometer scale [17]. The final cavity length in this research was 30.767 μm, which was calculated by the ratio λ_1_λ_2_/2(λ_1_ − λ_2_) (*λ*_1_ and *λ*_2_ are the adjacent peak wavelengths of the FP interference spectrum). Figure 7 shows the interference spectrum of the FP cavity as well as the superluminescent light emitting diode (SLED) spectrum.

### 3.2. Sensitivity and Resolution

**Figure 8:**
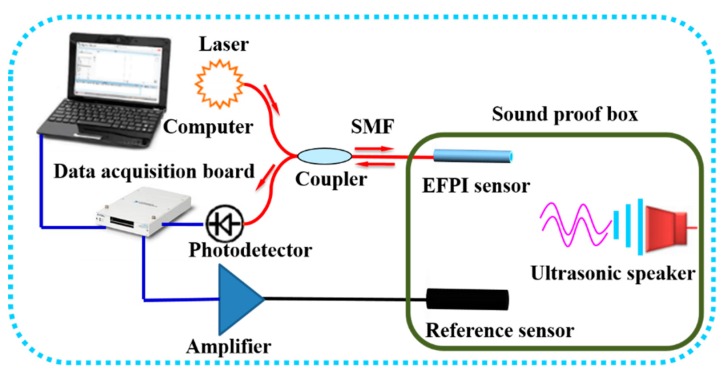
Experimental setup to characterize the fiber optic EFPI sensor system.

Figure 8 shows the experimental setup employed for the measurement of sensitivity and resolution. It was mainly composed of the fiber optic EFPI ultrasonic sensor, a data acquisition board, a computer, a reference sensor (sensitivity: 5 mV/Pa, amplifier gain: 100, frequency response: 20–70 kHz), and an ultrasonic speaker (frequency: 20–100 kHz). The final testing data were collected by the data acquisition board and processed by the computer. A DFB (distributed feedback) laser (wavelength: 1550 nm, power: 1 mW) was used as the light source of the sensing system, and the sensitivity and the amplifier gain of the photodetector were 0.9 A/W and 0.6 × 10^4^ V/A, respectively. The end reflectivity of the fiber optic PC was 4%, and the insertion losses of the fiber optic and the coupler were less than 0.15 dB.

**Figure 9:**
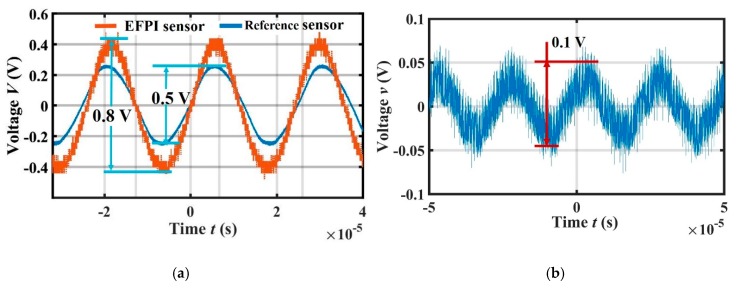
(**a**) Output peak voltages of the EFPI sensor and the reference sensor under the same ultrasonic pressure; (**b**) Minimum output voltage of the EFPI sensor system.

The same ultrasonic pressure generated by the ultrasonic speaker was applied to the reference sensor and the EFPI sensor, which were located in a symmetrical position relative to the ultrasonic generator. When the ultrasonic pressure of 40 kHz was applied, the output peak voltages of the EFPI sensor and the reference sensor were 0.8 V and 0.5 V, respectively, as shown in Figure 9a. According to the sensitivity and amplifier gain of the reference sensor, the ultrasonic pressure applied to both sensors was 1 Pa, and thus the sensitivity of the EFPI sensor was 0.8 V/Pa. When the ultrasonic pressure was reduced gradually, the minimum output peak voltage of the EFPI sensor was 0.1 V, as shown in Figure 9b. Since the output signal would be submerged by the voltage noise if the output peak voltage was less than 0.1 V, the minimum detectable pressure of the EFPI sensor for a 40 kHz ultrasonic wave is 0.13 Pa.

**Figure 10:**
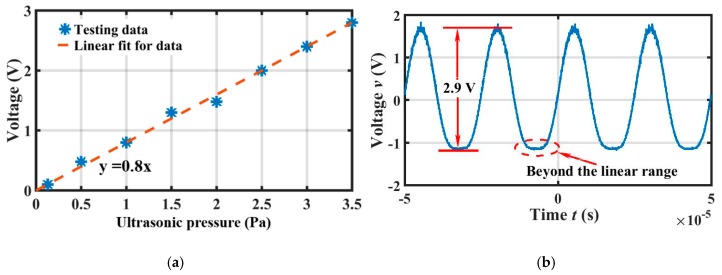
(**a**) Linearity of the sensor from 0.13 Pa to 3.5 Pa; (**b**) Output voltage of the sensor system when the diaphragm deformation exceeded the maximum linear range of FP interference.

When the output peak voltage was higher than 2.8 V with increasing ultrasonic pressure, there was a relatively straight line at its valley positions, as shown in Figure 10b. This indicated that the diaphragm deformation exceeded the maximum linear range of the FP interference, and in this region, light intensity changed very little with variations of the cavity length. When weak changes of light intensity could not be recognized by the photodetector, the output voltage became a straight line. As a result, the maximum output peak voltage of the EFPI sensor was 2.8 V, which indicates that the maximum detectable ultrasonic pressure was 3.5 Pa according to the output peak voltage of the reference sensor of 1.75 V, and the linearity of the sensor from 0.13 Pa to 3.5 Pa was 3.6%, as shown in Figure 10a.

**Figure 11:**
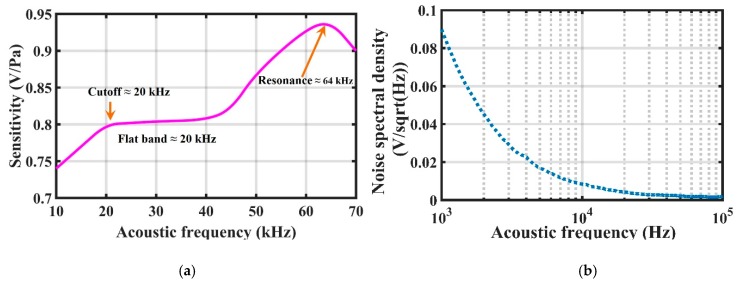
(**a**) Sensitivity of the sensor versus acoustic frequency; (**b**) Noise spectral density of the sensor.

Similarly, the sensor sensitivity versus ultrasonic frequency from 10 kHz to 70 kHz could be obtained by changing the emission frequency of the ultrasonic speaker (since the maximum detection frequency of the reference sensor was 70 kHz). The results showed that the flatband response was from 20 kHz to 40 kHz, and its sensitivity was around 0.8 V/Pa, as shown in Figure 11a. The resonant frequency was around 64 kHz; the small difference between the measured and the calculated resonant frequencies (60 kHz) might have been caused by the slightly different dimensions of the fabricated and modelled diaphragms. Figure 11b shows the noise spectral density of the sensor (in V/sqrt(Hz)), which belongs to 1/*f* noise, and the noise spectral density changes from 1.2 × 10^−3^ V/sqrt(Hz) to 0.5 × 10^−3^ V/sqrt(Hz) between 20 kHz and 40 kHz. The minimum detectable pressure can also be given by the noise spectral density of the sensor divided by the sensitivity (in V/Pa). In accordance to this, the minimum detectable ultrasonic pressure was 1.5 mPa/sqrt(Hz)–0.625 mPa/sqrt(Hz) between 20 kHz and 40 kHz. The background noise of the sensor was dominated by the optoelectronic noise. Therefore, reducing the background noise was a key measure to improve the performance of the sensor. For example, by using a better signal processor such as the one available from FISO Technologies, the performance might be further improved.

## 4. Conclusions

In this paper, a novel fiber optic EFPI ultrasonic sensor was designed, fabricated, and tested. The diaphragm of the fiber optic EFPI sensor was fabricated by using the MEMS processing technology on a SOI wafer. The diaphragm thickness was 5 μm, and the difficulty in controlling its desired thickness was greatly reduced by utilizing the proposed manufacturing procedure. In the batch production of the diaphragm, the FP cavity length was determined by a pre-etched stepped hole, which made the sensor assembly easier. Moreover, all the manufacturing processes of the diaphragm were simple and reliable. The described sensor is of relatively low cost, as it benefits from the MEMS fabrication technology and employs cheap and standardized components instead of expensive and customized ones. The final testing result showed that the minimum detectable ultrasonic pressure was 1.5 mPa/sqrt(Hz)–0.625 mPa/sqrt(Hz) between 20 kHz and 40 kHz. In addition, the sensing performance can be further improved by choosing a SOI wafer with a thinner device thickness and a further optimized structural design. This flexibility can expand the application fields of fiber optic EFPI ultrasonic sensors.

## Figures and Tables

**Table 1:** Characteristic material parameters of silicon and silicon nitride utilized in the design.

Material	Poisson’s Ratio *μ*	Elastic Modulus *E* (GPa)	Density *ρ* (kg/m^3^)
**Silicon**	0.22	163	2330
**Silicon Nitride**	0.25	310	3260
